# Luminescent manganese-doped CsPbCl_3_ perovskite quantum dots

**DOI:** 10.1038/srep45906

**Published:** 2017-04-12

**Authors:** Chun Che Lin, Kun Yuan Xu, Da Wang, Andries Meijerink

**Affiliations:** 1 Condensed Matter and Interfaces, Debye Institute for Nanomaterials Science, Utrecht University, Princetonplein 5, 3584 CC Utrecht, The Netherlands; 2 Soft Condensed Matter, Debye Institute for Nanomaterials Science, Utrecht University, Princetonplein 5, 3584 CC Utrecht, The Netherlands

## Abstract

Nanocrystalline cesium lead halide perovskites (CsPbX_3_, X = Cl, Br, and I) form an exciting new class of semiconductor materials showing quantum confinement. The emission color can be tuned over the full visible spectral region making them promising for light‒emitting applications. Further control over the optical and magnetic properties of quantum dots (QDs) can be achieved through doping of transition metal (TM) ions such as Mn^2+^ or Co^2+^. Here we demonstrate how, following QD synthesis in the presence of a Mn‒precursor, dropwise addition of silicon tetrachloride (SiCl_4_) to the QDs in toluene results in the formation of Mn‒doped CsPbCl_3_ QDs showing bright orange Mn^2+^ emission around 600 nm. Evidence for successful doping is provided by excitation spectra of the Mn^2+^ emission, with all features of the CsPbCl_3_ QD absorption spectrum and a decrease of the 410 nm excitonic emission life time with increasing Mn‒concentration, giving evidence for enhanced exciton to Mn^2+^ energy transfer. As a doping mechanism we propose a combination of surface etching and reconstruction and diffusion doping. The presently reported approach provides a promising avenue for doping TM ions into perovskites QDs enabling a wider control over optical and magnetic properties for this new class of QDs.

Lead halide perovskites MPbX_3_ (where M = CH_3_NH_3_^+^, HC(NH_2_)_2_^+^, and Cs^+^; X = Cl, Br, and I) have recently gained interest as the result of their promising performance for a variety of optoelectronic applications, especially photovoltaics^1^,^2^,^3^, but also light‒emitting diodes (LEDs)^4^,^5^, lasing^6^,^7^, and photodetectors^8^,^9^. These materials have favorable intrinsic properties including broad absorption spectra, tunable optical spectra, high luminescence quantum yields, high carrier mobility, and long carrier diffusion length. In the past three years, a rapidly increasing number of studies were published focused on synthetic methods and morphology control of colloidal perovskites nanocrystals (NCs) aimed at improving their performance in practical applications. The hybrid organic‒inorganic perovskites materials, such as CH_3_NH_3_PbX_3_ (X = Cl, Br, and I), are outstanding solar harvesting materials in photovoltaic devices with 20% certified power conversion efficiencies^10^,^11^. Sargent *et al*. utilized perovskites shell‒capped colloidal QDs films of (PbS‒CH_3_NH_3_PbI_3_) to fabricate novel and efficient photovoltaic devices^12^.

The all inorganic analogue perovskites CsPbX_3_ QDs were soon after reported by Kovalenko *et al*.^13^. These QDs show efficient luminescence that can be tuned over the full visible spectrum (400‒700 nm) by tuning of size (through quantum confinement effects) and composition (anion exchange)^13^,^14^. Using a facile solution‒based synthesis approach, cubic CsPbX_3_ QDs with colorful narrow emission bands and high quantum yields of up to 90% were realized^13^. The highly efficient narrow band emission makes these QDs very useful for application in lighting and displays. For example, colloidal CsPbBr_3_ QDs have been reported with efficient narrow band emission (FWHM = 86 meV; quantum yield = 90%) and reduced emission blinking making them promising for the lighting and display industry^15^. Interestingly, it is not generally known that this class of CsPbX_3_ QDs has been known for almost 20 years. Emission and quantum confinement of CsPbX_3_ QDs that form in Pb‒doped CsX scintillator crystals was reported in the late 1990’s^16^. For the II‒VI QDs such as ZnO, ZnS, ZnSe, CdS, CdSe there has been a strong effort to dope the QDs with transition metal (TM) ions such as Mn^2+^ or Co^2+^ aimed at introducing new optical and/or magnetic functionalities^17^,^18^,^19^,^20^,^21^. A large number of publications have reported different categories of doped QDs^22^,^23^,^24^. For example, efficient Mn^2+^ emission in ZnSe QDs is promising for application in solar concentrators as a result of the large spectral shift of the Mn^2+^ emission which prevents undesired reabsorption^25^. Doping of magnetic ions in QDs also holds great promise in the field of spintronics with the possibility to dope a single magnetic ion in a single QD.

Doping can be straightforward by simply adding a dopant precursor in the reaction mixture, e.g. in ZnS:Mn^2+^, but more often doping of QDs proved to be challenging^26^. In the past decade new doping techniques have been developed allowing doping of systems that were previously considered not possible to dope. For example, doping CdSe with Mn^2+^ was considered impossible but was later realized using a single source precursor method. More recently with diffusion doping even concentrations up to 30% Mn^2+^ are now incorporated in CdSe^27^. For perovskite QDs doping has only recently been realized and is highly interesting as it allows the addition of optical or magnetic functionalities to this exciting new class of materials^28^,^29^.

Here we report doping of Mn^2+^ into perovskite CsPbCl_3_ QDs (CPC‒Mn) via a facile colloidal approach. A post synthesis treatment of CsPbCl_3_ QDs prepared in the presence of Mn‒stearate allows incorporation of Mn^2+^. Slowly dripping a small volume (5‒20 μl) of SiCl_4_ to the dispersion of QDs in toluene results in a slow incorporation of Mn^2+^ at room temperature. Evidence for successful doping is provided by a clear signature of efficient energy transfer from the CsPbCl_3_ QD exciton to Mn^2+^ ions. As Mn^2+^ incorporation proceeds, the excitonic emission intensity decreases and the bright orange Mn^2+^ emission increases. The excitation spectrum of the Mn^2+^ emission shows all the features of the CsPbCl_3_ QD exciton absorption. The life time of the exciton emission decreases as the Mn^2+^ concentration increases as a result of fast exciton to Mn^2+^ energy transfer. The proposed mechanism for incorporation involves surface etching and reconstruction and diffusion doping. Decomposition of SiCl_4_ by trace amounts of water in the toluene yields HCl. Surface etching by H^+^ and surface reconstruction enabled by chloride ions and Mn^2+^ form a surface layer and slowly drives Mn^2+^ into the QDs by ion diffusion. The decomposition of SiCl_4_ also gives rise to the formation of a protective SiO_2_ layer giving a relatively high stability to the QDs. Perovskites QDs are known to degrade and show a fast loss of emission intensity under ambient conditions. The PL of the CPC‒Mn QDs synthesized is relatively stable and retains over 25‒30% of the maximum intensity after stirring with SiCl_4_ for 40 days.

## Results and Discussion

### Synthesis and characterization

CsPbCl_3_ QDs were prepared using a similar procedure as reported by Kovalenko *et al*.^13^. Details on the QD synthesis and doping procedure can be found in the Methods section. In short, PbCl_2_ and the Mn‒precursor (Mn‒stearate) in 1‒octadecene (ODE) were dried at 120 °C after which dried oleylamine (OLAM) and oleic acid (OA) were added. The temperature was raised to 170 °C and a Cs‒oleate solution in ODE was injected. At this temperature the CsPbCl_3_ QDs form quickly. Next, the mixture was cooled and the NCs were extracted by centrifugation. To remove unreacted polar material the QD precipitate was redispersed in toluene and centrifuged. Precipitated particles were discarded. The final solution of CsPbCl_3_ QDs in toluene shows the characteristic violet QD emission but no Mn^2+^ emission, indicating that in this procedure Mn^2+^ is not incorporated. In the next step a small volume (5‒20 μl) of SiCl_4_ was dripped to 2 ml of the QD solution. During stirring a white precipitate forms. After different stirring times the precipitate was removed by centrifugation and a transparent QD solution was used for the spectroscopic measurements and characterization. As a control experiment SiCl_4_ was also added under vacuum conditions in which all water was removed from the toluene.

**Figure 1:**
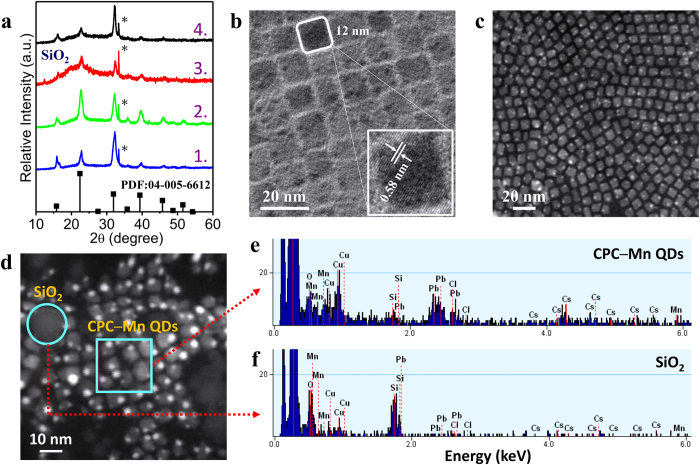
Characterization of CPC‒Mn QDs. (**a**) X‒ray diffractograms of as synthesized CsPbCl_3_ (1), CsPbCl_3_ with added Mn^2+^‒precursor (2), CsPbCl_3_ with Mn^2+^‒precursor after addition of SiCl_4_ and stirring for 1 h (3) and 10 days (4) compared to the standard powder diffraction pattern (black vertical lines) of cubic bulk CsPbCl_3_ (* marks extra peaks are caused by the silicon wafer). (**b**) TEM image of the CPC‒Mn QDs. The inset shows a higher magnification image of a cubic particle. (**c**) HAADF‒STEM image of the CPC‒Mn QDs (after centrifuging) with SiCl_4_ and stirring 10 days. (**d**) HAADF‒STEM image of the CPC‒Mn QDs (solution without centrifuging) with SiCl_4_ for stirring 10 days. (**e,f**) EDX analysis for a CPC‒Mn QDs region (cyan square) and a region without QDs (cyan circle) from Fig. 1d.

In Fig. 1a X‒ray diffraction (XRD) patterns of the various synthesis products are shown. Line 1 (blue) shows the typical XRD pattern of CsPbCl_3_ QDs made without Mn^2+^. The XRD pattern of the QDs synthesized in the presence of the Mn‒stearate precursor (green line) is similar. For the product formed after addition of SiCl_4_ and stirring for 1 h (red line) the formation of amorphous SiO_2_ is evident from the broad diffraction peak of amorphous SiO_2_ (PDF Card No. 82‒1576), observed clearly from 15° to 30°. Stirring for 10 days, the pure CsPbCl_3_ XRD patterns (black line) was still found when the precipitate (SiO_2_) was removed by centrifuging. The lines in the XRD patterns of the QDs match the powder diffraction pattern of cubic phase CsPbCl_3_ (Fig. S1a). The broadening of the diffraction peaks is because of the small size. The TEM image (Fig. 1b) shows the formation of monodisperse (13.1 ± 0.4 nm) side length CPC‒Mn QDs and a d‒spacing of 0.58 nm is clearly observed^13^,^30^. The particle size is similar to that for samples made without Mn‒precursor (Fig. S2). Figure S3 displays elemental analysis by energy dispersive X‒ray spectroscopy (EDX) to confirm that Mn is present in the sample after doping, although no Mn^2+^ emission is observed. Given the highly apolar nature of the Mn‒precursor, the Mn‒stearate can be expected to absorb in the apolar capping layer around the QDs.

In the next step a small volume of SiCl_4_ is added to the QDs in toluene. The role of this silica precursor is twofold: it will induce the growth of protective SiO_2_ around the QD, and, more importantly, it induces the incorporation of Mn^2+^ in the CsPbCl_3_ QDs. The role of SiO_2_ as a protective shell to protect the core material is well‒known. For example, the stability of CH_3_NH_3_PbBr_3_‒SiO_2_^31^, CdSe/ZnS‒SiO_2_^32^, and CdSe/CdS/ZnS‒SiO_2_^33^, QDs were clearly improved in comparison to the uncoated QDs. The High Angle Annular Dark Field Scanning TEM (HAADF‒STEM) image after addition of SiCl_4_ and stirring for 10 days and removal of SiO_2_ by centrifugation is shown in Fig. 1c. The monodisperse cubic particles (7.7 ± 0.5 nm) are smaller than that for the QDs before SiCl_4_ addition. This indicates that SiCl_4_ addition causes etching of the CsPbCl_3_ QDs. In the HAADF‒STEM images also white dots are observed. Elemental mapping (Fig. S4) reveals that these dots are highly enriched in Pb.

In the HAADF‒STEM image of the sample before centrifugation both individual CPC‒Mn QDs and SiO_2_ nanoparticles were observed (Fig. 1d). This indicates that also a secondary nucleation of silica occurred and that silica was not only deposited on the QD surface in a seeded growth. EDX analysis for a region containing CPC‒Mn QDs (cyan square Fig. 1d) and a region containing mainly SiO_2_ (cyan circle Fig. 1d) are shown in Fig. 1e and f. In the EDX spectrum of the QD area (cyan square) signals of Cs, Pb, Cl, and Mn of the CPC‒Mn QDs are observed and signals corresponding to Si and O are weak. The elemental analysis of the cyan circle shows strong Si and O signals, indicating the presence of a SiO_2_ nanoparticles.

In a control experiment, SiCl_4_ was added to the QD dispersion and then stirred for 3 days in vacuum. TEM image and EDX analysis were recorded for the CPC‒Mn QDs made under vacuum are shown in Fig. S5. The CPC‒Mn QDs were covered by a SiO_2_ layer (Fig. S5a). The different SiO_2_ morphology is ascribed to a difference in water content which determines the HCl concentration following hydrolysis of SiCl_4_^34^,^35^. The water present in toluene under ambient conditions (H_2_O content ~0.06%) induces faster decomposition of SiCl_4_ and silica formation^31^. In the resulting SiO_2_ covered CPC QD conglomerates all elemental signals of the CPC‒Mn QDs and SiO_2_ (Cs, Pb, Cl, Mn, Si, and O) were observed, as shown in Fig. S5b.

### Optical spectroscopy

**Figure 2:**
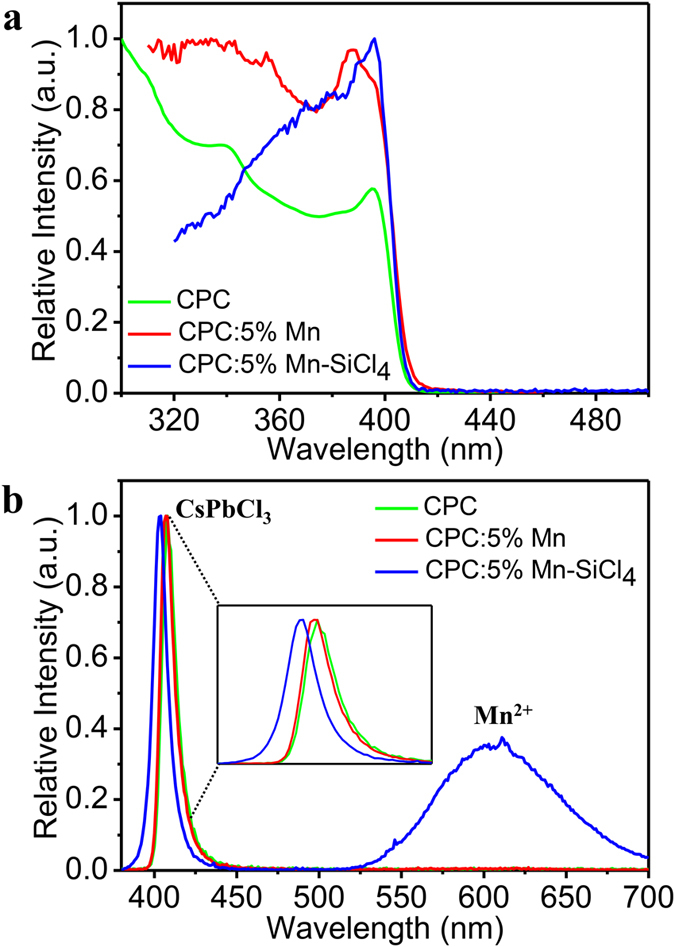
Optical properties of the CsPbCl_3_:5%Mn^2+^ QDs. (**a**) Absorption spectrum (green line) of undoped CsPbCl_3_ NCs. Excitation spectra of the CsPbCl_3_:5%Mn^2+^ (red line) and CsPbCl_3_:5%Mn^2+^ after SiCl_4_ addition and stirring for 1 day (blue line). (**b**) Emission spectra of the undoped CsPbCl_3_ (green), CsPbCl_3_:5%Mn^2+^ (red) and CsPbCl_3_:5%Mn^2+^ QDs after SiCl_4_ addition and 1 day stirring (blue).

To monitor incorporation of luminescent dopants into QDs optical and time resolved spectroscopy are powerful tools. When a luminescent ion is inside of a QD, fast energy transfer from the QD exciton to the dopant is expected. Absorption of light by the QD is followed by exciton‒to‒dopant energy transfer and emission from the dopant ion. Monitoring emission and excitation spectra as well as changes in the time traces of the luminescence intensity of excitonic and dopant emission provides clear signatures for the successful incorporation of a dopant in a QD. In Fig. 2a,b absorption, excitation, and emission spectra are shown for three types of CsPbCl_3_ QDs: undoped QDs (CPC, no Mn added, green lines), CsPbCl_3_:5%Mn QDs (CPC:5%Mn, 5% Mn‒precursor added to reaction mixture, red lines) and CsPbCl_3_:5%Mn‒SiCl_4_ (CPC:5%Mn‒SiCl_4_, 5% Mn‒precursor added, followed by 10 μl SiCl_4_ and stirring for 1 day, blue lines). All three QDs show exciton emission around 400~410 nm. The emission spectra of the CPC and CPC:5%Mn samples are almost identical. For the CPC:5%Mn‒SiCl_4_ sample an extra emission band is observed in the red/orange spectral region around 600 nm. The excitonic emission shows a small (5 nm) blue shift and is slightly broader (halfwidth = 600 cm^−1^ versus 550 cm^−1^ for the CPC and CPC:5%Mn‒SiCl_4_ exciton emission).

The 600 nm emission wavelength is typical of the ^4^T_1_ → ^6^A_1_ emission from Mn^2+^. Mn^2+^ can emit in the green to deep red spectral region depending on the local coordination. The Tanabe‒Sugano diagram for ions with the 3d^5^ configuration shows that the position of the emitting lowest energy excited state ^4^T_1_ shifts to lower energies as the crystal field splitting increases^36^,^37^. In CsPbCl_3_ the Mn^2+^ will substitute on the octahedrally coordinated Pb^2+^ site (the Cs^+^‒site is too large). Since Cl^-^ ions are weak crystal field ligands in the spectrochemical series a green emission may be expected. However, octahedral coordination gives rise to a large crystal field splitting and even for weak ligands orange‒red emission is typically observed for Mn^2+^. For example, in CdCl_2_ and a variety of chloride perovskites where Mn^2+^ is in an octahedral Cl^-^ coordination, the emission maximum of the ^4^T_1_‒^6^A_1_ emission of Mn^2+^ has been observed at wavelengths varying between 570 nm and 630 nm^36^,^37^. The presently observed emission maximum at 600 nm is in excellent agreement with this range and provides further support for the assignment of the orange emission to ^4^T_1_‒^6^A_1_ emission of Mn^2+^ in an octahedral Cl^-^ coordination.

The absorption and excitation spectra of the different CPC QDs in Fig. 2a are all similar and show an exciton peak at 410 nm. Interestingly, the excitation spectrum of the red/orange Mn^2+^ emission for the CPC:5%Mn‒SiCl_4_ QDs (blue line) also shows the excitonic features of the QD absorption, indicating exciton‒to‒Mn^2+^ energy transfer, a signature of incorporation of Mn^2+^ in the CsPbCl_3_ QD. Careful inspection of the emission spectra for CPC:5%Mn before SiCl_4_ addition reveals a very weak emission feature in the orange spectral region (Fig. S6) that is not observed for the undoped CPC sample (Fig. 2b, green line). The weak Mn^2+^ emission disappears after washing with acetone (see Fig. S7) and also no Mn^2+^ emission was observed for acetone‒washed CPC:5%Mn after addition of SiCl_4_ under ambient conditions. This result indicates that the weak emission originates from Mn^2+^ precursor adsorbed in the QD surface layer. As the Mn^2+^ ions are not incorporated in the QD, energy transfer from the exciton to Mn^2+^ excited states is not efficient due to the weak coupling of the exciton to remote Mn^2+^ ions. Washing with acetone removes the surface absorbed Mn^2+^ precursor.

As shown above, the addition of a small volume of SiCl_4_ to the CPC QDs in toluene gives rise to Mn^2+^ emission after prolonged stirring at room temperature. To study the influence of the volume of SiCl_4_ added, emission spectra were recorded after addition of 5~20 μl SiCl_4_ into the CPC:5%Mn QDs toluene solution under ambient conditions. The emission spectra, shown in Fig. S8, show an enhanced CPC QD exciton emission as well as Mn^2+^ emission for all solutions after 12 h of stirring following SiCl_4_ addition. The increase in PL intensity is explained by better surface passivation of the QDs and will be discussed below. The intensity of the Mn^2+^ emission increases for SiCl_4_ volumes up to 10 μl and then decreases. For the highest volumes added (15 or 20 μl SiCl_4_) degradation of the CsPbCl_3_ QDs was observed. As the stability of the CPC QDs is compromised, also the Mn^2+^ emission intensity decreased for the highest volumes of SiCl_4_ added. For the reaction volume used (2 ml), the optimum SiCl_4_ amount equals 10 μl and this volume was used in subsequent experiments to follow the time evolution of Mn^2+^ incorporation.

**Figure 3:**
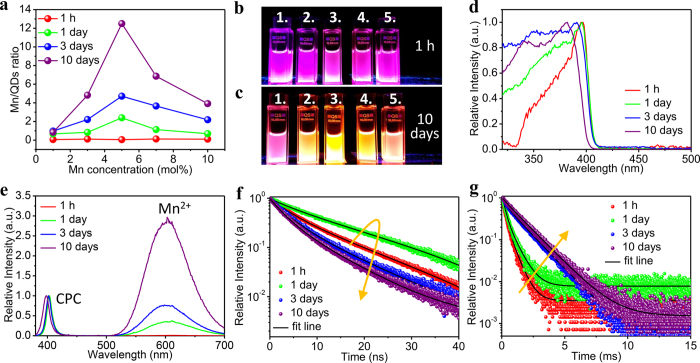
Spectral evolution of CsPbCl_3_:x%Mn^2+^ (x = 1, 3, 5, 7, and 10) QDs and CsPbCl_3_:5%Mn^2+^ QDs as function of reaction time. (**a**) Mn^2+^/Exciton emission ratio for different reaction times from 1 h to 10 days. Photographs of QD solutions under UV illumination after stirring for 1 h (**b**) and 10 days (**c**). Numbers (1→5) represent different Mn^2+^ concentration (1→10 mol%). (**d**) Excitation spectra and (**e**) emission spectra of CsPbCl_3_:5%Mn^2+^ QDs with 10 μl SiCl_4_. PL decays of CsPbCl_3_ QD exciton emission (~400 nm) (**f**) and Mn^2+^ emission (~600 nm) (**g**) after 376.8 nm and 355 nm pulsed excitation.

To optimize the Mn‒concentration and to probe Mn‒incorporation over time, extensive spectroscopic characterization was done for CPC:x%Mn‒SiCl_4_ samples taken at different times after injection of SiCl_4_. In Fig. 3 excitation and emission spectra, luminescence decay curves of the excitonic and Mn^2+^ emission as well as the integrated emission intensity ratios of Mn/QD are shown for different times (1 hour to 10 days) after injection of 10 μl SiCl_4_ to a CPC:x%Mn QD dispersion prepared with 1, 3, 5, 7 and 10% of Mn‒precursor present in the initial reaction (concentrations in mole% relative to Pb^2+^). Figure 3a shows the ratio of the Mn^2+^ to QD emission intensity for samples taken after 1 h to 10 days. In Fig. 3b,c photographs are shown of the QD dispersions under UV (365 nm) illumination after 1 h and 10 days of stirring. In the photographs a clear change in emission color is observed between 1 h and 10 days. As a result of increased Mn‒incorporation the emission color shifts from violet to orange. Interestingly, the Mn/QD intensity ratios drastically increase after stirring for 1 day (green line). At all times, the highest Mn/QD ratio is found for a 5%Mn concentration. For higher nominal Mn-concentrations a drop in emission intensity is observed, possibly caused by concentration quenching. Concentration quenching is caused by energy transfer between dopant ions leading to migration to defects and quenching sites. Typically, this occurs at concentrations higher than 5% but as the distribution of the Mn^2+^ ions may not be homogeneous, a higher concentration of Mn^2+^ ions in the surface layer can explain a reduced luminescence intensity by concentration quenching through energy migration in a Mn-enriched surface layer. Based on this, it is concluded that the reaction conditions leading to CPC‒Mn QDs with the highest Mn‒emission intensity is CPC:5%Mn with 10 μl SiCl_4_ for the reaction volume (2 ml) and QD concentrations (20 mg/ml) used here. The changes in luminescence properties over time were followed for the CPC:5%Mn‒SiCl_4_ (10 μl) by recording excitation and emission spectra as well as luminescence decay curves of the exciton and Mn^2+^ emission for samples taken after 1 h, and 1, 3, 10 days. The results are shown in Fig. 3d‒g.

Focusing on the CPC:5%Mn‒SiCl_4_ (10 μl) system, the results show a continued increase in the Mn^2+^ emission intensity relative to the excitonic emission (Fig. 3e) for reaction times up to 10 days. The excitation spectra of the Mn^2+^ emission show the characteristics of the CsPbCl_3_ QD absorption spectrum, giving evidence for energy transfer from the QD exciton state to the Mn^2+^ dopant. For longer reaction times a blue shift in the first excitonic peak is observed, both in emission and excitation (Fig. 3d‒e). The shift to shorter wavelengths can be explained by a decrease in the size of the QDs as a result of slow etching accompanying the Mn‒incorporation process. As shown in Fig. 1 the QD size decreases from 12 to 8 nm after 10 days of stirring and this can explain the observed blue shift. The highest Mn^2+^ emission intensity is observed after 10 days of stirring. At longer times the intensity starts to decreases but even after 40 days a clear Mn^2+^ emission is observed with ~30% of the maximum emission intensity.

To gain further insight in the energy transfer process and incorporation of Mn^2+^ in the CPC QDs, luminescence decay curves were recorded for both the exciton and Mn^2+^ emission. Table 1 summarizes the lifetime (for QD exciton and Mn^2+^ emission) of CPC:5%Mn‒SiCl_4_ (10 μl) for various reaction times. Energy transfer from the excitonic state to Mn^2+^ results in a faster exciton emission decay. The Mn^2+^ emission decay can be different for surface absorbed Mn^2+^ than for Mn^2+^ incorporated in the QD as energy transfer to defects or surface quenching sites is more prominent for surface Mn^2+^ resulting in shorter decay times for emission from surface Mn^2+^ ions. The luminescence decay dynamics were recorded for samples taken at several times during the incorporation of Mn^2+^ for the CPC:5%Mn‒SiCl_4_ (10 μl) system as this was shown to give the most efficient Mn^2+^ emission. In Fig. 3f, the PL decay curves of the excitonic QD emission are shown. Initially, *i.e.* for the decay curves recorded for samples taken after 1 h to 1 day, a lengthening of the decay time is observed. The decay curve for the exciton emission from the sample stirred for 1 day is close to single exponential with a ~16 ns decay time. The initial lengthening of the decay time can be explained by improved surface passivation as a result of the chloride formation following SiCl_4_ decomposition. Better surface passivation by the Cl^-^ ions remove surface trap states resulting in a more efficient excitonic emission. The 16 ns decay time can be considered to be close to the pure radiative decay time of the exciton emission at room temperature. The results agree with the observation of higher absolute (exciton) emission intensities of CPC QDs in the initial reaction period (up to 1 day) as shown in Fig. S8a.

After prolonged reaction times, the Mn^2+^ emission intensity strongly increases, while the excitonic emission intensity decreases. Concurrently, the exciton emission decay becomes shorter and also non‒exponential (blue and violet curves in Fig. 3f). After stirring for 10 days a τ_avg_ = 6.3 ns decay time is observed. The shortening of the decay time is explained by energy transfer from the QD exciton state to the Mn^2+^ dopants. The non‒exponential character reflects that transfer rates vary for differently doped QDs. Undoped QDs still decay with the radiative exciton decay time while QDs with one or more Mn^2+^ ions will decay with a decay rate that is the sum of the radiative decay rate and the transfer rate. The exciton‒to‒Mn^2+^ transfer rate will also depend on the location of the Mn^2+^ ion in the CPC‒Mn QD. Transfer to a centrally located Mn^2+^ ion is expected to be faster than to a Mn^2+^ ion in the outer shell of the QD where the overlap with excitonic wavefunction is smaller.

Figure 3g show the luminescence decay curves of the Mn^2+^ emission as a function of reaction time. Long decay times in the ms time regime are observed which is typical for the spin‒ and parity forbidden Mn^2+^ emission^23^,^24^. Initially the decay curves are non‒exponential and the faster initial decay reflects emission from Mn^2+^ at the QD surface where partial quenching by surface defect states introduces a fast non‒radiative decay channel. For long reaction times (3 and 10 days) the decay is close to single exponential with a long 1.4 ms decay time suggesting that Mn^2+^ is incorporated inside the CPC host and decays with a ms radiative decay rate typical for the spin‒ and parity forbidden Mn^2+^ emission.

### Reaction mechanism

The synthesis procedure and characterization discussed above provide strong evidence that addition of SiCl_4_ to the CPC QDs in toluene in the presence of the Mn‒stearate precursor results in the slow incorporation of Mn^2+^ in the CsPbCl_3_ QDs. It is interesting to discuss potential incorporation mechanisms and the location of the Mn^2+^ ions in the CPC QDs. Control experiments help to provide insight in the mechanism. No incorporation of Mn^2+^ was observed after thorough washing of the CPC‒Mn QDs with acetone before the addition of SiCl_4_ (Fig. S7). This shows that the (apolar) Mn‒precursor is absorbed at the CPC QD surface after the initial QD synthesis procedure in the presence of the Mn‒stearate precursor. A second control experiment involves dripping of SiCl_4_ to the CPC QDs with Mn‒precursor in toluene in vacuum. Again, no Mn‒incorporation is observed under these conditions. Only the weak emission of Mn^2+^ ions around 590 nm and a faint blue emission, possibly from organic chromophores formed during ligand decomposition, were observed (Fig. S9). The absence of water prevents decomposition of SiCl_4_ and demonstrates that hydrolysis of SiCl_4_ is crucial in the reaction:


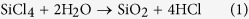


**Figure 4:**
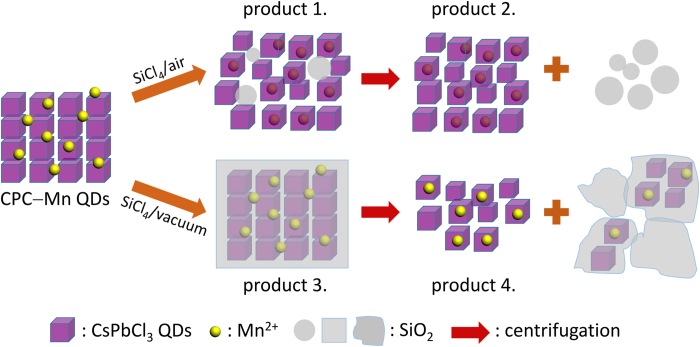
Schematic illustration of Mn^2+^ ions diffusion doping mechanism and SiO_2_ formation. Purple cubes represent the CsPbCl_3_ QDs; the yellow spheres represent the Mn^2+^ ions; the gray particles represent SiO_2_; and the red arrows represent centrifugation.

Hydrolysis of SiCl_4_ gives rise to the formation of HCl that can react with the Mn‒stearate precursor (releasing Mn^2+^) and with the CPC QD surface to both etch the surface (H^+^) and reconstruct the QD surface (Cl^-^). The blue shift of the excitonic emission with reaction time and the TEM images showing a decrease in particle size are consistent with the occurrence of etching of the CPC QDs. The SiO_2_ formed during the decomposition reaction of SiCl_4_ and H_2_O gives rise to SiO_2_ spheres observed in TEM and possibly also form a thin protective SiO_2_ layer around the CPC QDs after prolonged reaction times. The increase of the QD exciton emission and lengthening of the exciton emission decay time in the initial hours after addition of SiCl_4_ signifies the positive role of the HCl in the passivation of surface defect states. Similar observations were made previously for hybrid perovskite thin films^38^. A remarkable increase in photoluminescence intensity and lengthening of the emission decay time was observed upon addition of phosphoric acid and explained by the removal of (surface) defect states^38^. In addition to surface reconstruction, in the presence of Mn^2+^ and Cl^-^ a Mn‒Cl surface layer can form on the CPC QD. Etching and reconstruction of the CsPbCl_3_ surface can occur with the incorporation of Mn^2+^ on Pb^2+^ sites in the surface layer of the CPC QDs. Subsequent diffusion of the Mn^2+^ into the CPC QD brings Mn^2+^ inside the CPC QDs, similar to the Mn^2+^ doping of CdSe QDs through diffusion doping^27^. Fig. 4 illustrates the proposed reaction mechanism for the incorporation of Mn^2+^ and SiO_2_ formation. The reaction mechanism is consistent with the long reaction times of hours to days observed for diffusion doping and cation exchange reactions at room temperature^18^,^39^.

Further research is required to provide a more complete understanding of details on the incorporation mechanism. Based on the present experiments it cannot be excluded that the Mn^2+^ ions remain located in a surface layer of the CPC QDs. The observation of a transition from non‒exponential decay to a single exponential 1.4 ms decay of the Mn^2+^ emission is important as it gives evidence that the local surroundings for the Mn^2+^ ions changes during the reaction. Initially surface Mn^2+^ ions are present with a shorter life time because of the proximity of surface defect states. For longer reaction times the transition to a purely exponential decay indicates the transition to the same (octahedral) coordination by Cl^-^ for all Mn^2+^ ions. This observation however does not prove that the Mn^2+^ ions are homogeneously distributed through the CsPbCl_3_ QD. The continued observation of exciton emission indicates that the transfer efficiency is limited and that the Mn^2+^ ions may be preferentially situated in the surface layer where the coupling with the excitonic wavefunction is weaker. Further research on incorporation of Mn^2+^ in other perovskite QDs is also interesting. For example, for CsPbBr_3_ QDs the exciton shifts to lower energies (compared to CsPbCl_3_) while, because of a smaller crystal field splitting for the weaker Br^-^ ligands, the Mn^2+^ emission is expected to shift to higher energies. This may lead to a cross-over between the exciton and Mn^2+^ emission depending on the quantum dot size, similar to what has been observed for Mn^2+^-doped CdSe quantum dots.^24^

### Stability

**Figure 5:**
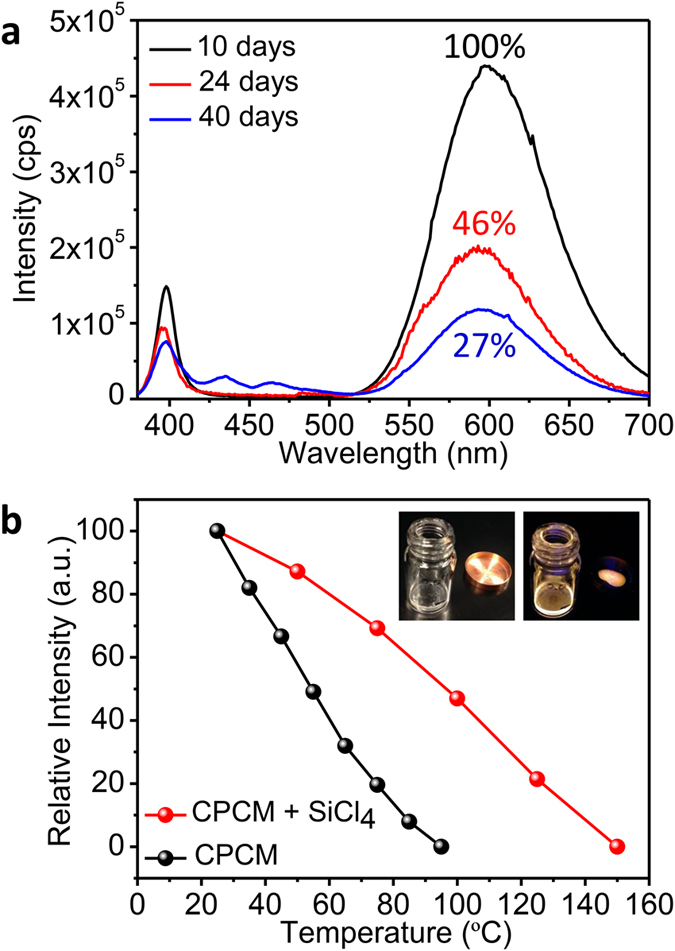
Stability of the CsPbCl_3_:5%Mn^2+^‒SiCl_4_ (10 μl) system. (**a**) Emission spectra recorded after different stirring times in air. (**b**) Relative emission intensity as a function of the temperature for the Mn^2+^ emission in CPC‒Mn‒SiCl_4_ (red line) and the exciton emission for the CPC QDs without SiCl_4_ added. [Inset: photographs of the CPC:5%Mn‒SiCl_4_ QDs in ambient light (left) and under irradiation with a UV lamp (right). The CPC:5%Mn‒SiCl_4_ QDs are shown in a vial with PMMA and the resulting PMMA‒QD polymer film on the copper heating block.] All measurements are done under 365 nm excitation.

For practical application of perovskite quantum dots stability is an important issue. Thermal and photo stability are often limited for the Pb‒halide perovskites and also sensitivity to moisture is serious issue that hampers the introduction of this new class of materials in products for the solar cell and lighting market^40^,^41^,^42^. To evaluate the stability of the emission of the SiCl_4_‒modified CPC‒Mn QDs solutions was followed over time. The emission intensity shows a slow decrease in intensity but retains 27% of the maximum intensity after 40 days in air, as shown in Fig. 5a. The temperature dependence of the photoluminescence was measured for CPC‒Mn QDs (exciton emission) and SiCl_4_‒modified CPC‒Mn QDs (Mn^2+^ emission) after incorporation in a poly methyl methacrylate (PMMA) matrix. The decrease of emission intensity with temperature is shown in Fig. 5b. At 100 °C the emission of CPC‒Mn QDs is completely quenched, whereas the SiCl_4_‒modified CPC‒Mn QDs maintained ~50% of their initial intensity.

In conclusion, we have reported a new doping strategy for the successful incorporation of Mn^2+^ ions in perovskite CsPbCl_3_ QDs. Upon addition of SiCl_4_ to a CsPbCl_3_ QD/Mn‒precursor dispersion in toluene incorporation of Mn^2+^ occurs as demonstrated by the evolution of strong 600 nm Mn^2+^ emission (^4^T_1_ → ^6^A_1_) following excitation in the QD exciton states. Excitation, emission and luminescence life time measurements reveal the presence of energy transfer from the delocalized QD exciton state to the localized 3d^5^ states of Mn^2+^. The proposed incorporation mechanism involves surface etching and reconstruction by HCl formed through decomposition of SiCl_4_ in the presence of trace amounts of water in toluene. The presently reported synthesis approach is promising for exploring other transition‒metal or lanthanide ion doped perovskite QDs in order to gain a wider control over the optical and magnetic properties of doped QDs for applications in the fields of lighting and spintronics.

## Methods

### Preparation of Cs‒oleate

The synthesis approach has been reported by Kovalenko *et al*.^13^. In brief, Cs_2_CO_3_ (0.2035 g, Sigma Aldrich, 99.9%), oleic acid (0.625 ml, OA, Sigma Aldrich, 90%), and 1‒octadecene (10 ml, ODE, Sigma Aldrich, 90%) were loaded into a 50 ml 3‒neck flask and degassed under vacuum at 120 °C for 1 h. Subsequently, the solution was heated to 150 °C under N_2_ for all Cs_2_CO_3_ reacting with OA.

### Preparation of CsPb_1‒x_Cl_3_:x%Mn^2+^ NCs

PbCl_2_ (0.0497 g, Sigma Aldrich, 99.999%), C_36_H_70_MnO_4_ (x = 5; 0.0058 g, Chemos GmbH), and ODE (5 ml) were loaded into a 25 ml 3‒neck flask and degassed under vacuum at 120 °C for 1 h. Afterwards, dried oleylamine (0.5 ml, OLA, Sigma Aldrich, 70%) and OA (0.5 ml) were injected into the solution at 120 °C under N_2_ atmosphere. The temperature was raised to 170 °C and Cs‒oleate solution (0.4 ml) was quickly injected into the mixture. After the desired different reaction time, the mixture was cooled by the ice‒water bath. The NCs were extracted from the crude solution through centrifuging at 3500 r.p.m. for 10 min. First, the supernatant was discarded and the precipitated particles were redispersed in toluene forming the stable colloidal solution. Second, the colloidal solution was centrifuged again, and the precipitated particles were discarded and the supernatant was redispersed in toluene forming the final solution.

### Addition of silicon tetrachloride (SiCl_4_)

Various amounts of SiCl_4_ (Sigma Aldrich, 99%) were introduced into a 20 ml sample bottle containing 2 ml of the colloidal CPC‒Mn QDs toluene solution (H_2_O content 0.0623%)^31^. After different stirring time, the white precipitates (SiO_2_) and the transparent solutions were separated through centrifuging at 10000 r.p.m. for 10 min. In a control experiment, 10 μl SiCl_4_ was mixed with CPC‒Mn QDs (nearly water free) in a vacuum three‒necked flask.

### Preparation of CsPbCl_3_:Mn^2+^ bulk material

The CPC‒Mn microcrystalline powder was prepared by melting starting material in an evacuated and sealed quartz ampoule^43^. High‒purity CsCl (Brunschwig Chemie B.V., 99.9%), PbCl_2_ (Sigma Aldrich, 99.999%), and MnCl_2_ (Sigma Aldrich, 99%) were mixed thoroughly and loaded into a quartz ampoule. The ampoule was evacuated and suspended with platinum wire in the high temperature region of a sintering furnace. The temperature was raised to 300 °C with a heating rate of 5 °C/min and kept for 2 h under continuous pumping to remove surface absorbed water. Then, the ampoule was sealed and the sample was sintered at 675 °C for 1 h, after which the sample was allowed to naturally cool to room temperature in the furnace.

### Polymerization reaction of CPC:5%Mn‒SiCl_4_ + PMMA

PMMA (0.8 g) powder was dispersed homogeneously in toluene (2 ml) for stirring 1 day. Then CPC:5%Mn‒SiCl_4_ QDs (20 mg) were added into the PMMA‒toluene solution and stirred for 1 day. The transparent mixture was dripped into a glass container and a copper hold, as shown in the inset of Fig. 5b. The polymerization took place under a vacuuming procedure for 1 day.

### X‒ray diffraction

XRD patterns were recorded using Cu Kα radiation (λ = 1.5418 Å) on a PW 1729 Philips diffractometer, operating at 40 kV and 20 mA. For XRD analysis, the samples were prepared by depositing the QDs colloidal solution on the silicon wafer and evaporating the solvent under vacuum atmosphere.

### Optical Characterization

Absorption spectra were obtained using a Perkin‒Elmer Lambda 950 UV/VIS/IR spectrophotometer. Excitation and emission spectra were measured by an Edinburgh Instruments FLS920 spectrofluorometer equipped with a 450 W Xenon lamp. Visible emission (400‒850 nm) was detected by a Hamamatsu R928 photomultiplier tube (PMT).

### Lifetime

Photoluminescence (PL) decay curves of the CsPbCl_3_ QDs were recorded by using an Edinburgh Instruments diode laser with an excitation wavelength of 376.8 nm (65 ps pulse width, 0.2‒20 MHz repetition rate) and a Hamamatsu H74220‒02 PMT in combination with the Edinburgh spectrofluorometer. PL decay measurements of the Mn^2+^ dopants were done using an optical parametric oscillator (OPO) system (Opotek HE 355 II) pumped by the third harmonic of a Nd:YAG laser as excitation source and a Hamamatsu R928 PMT as light detection. The samples for optical analysis were prepared by dissolving the crude QDs production in toluene in a quartz cuvette.

### Electron Microcopy and Energy Dispersive X‒ray Spectroscopy

Scanning transmission electron microscopy (STEM) images and Energy dispersive X‒ray spectroscopy (EDX) measurements were performed using a Talos^TM^ F200X transmission electron microscope (from FEI), equipped with a high‒brightness field emission gun (X‒FEG) and a Super‒X G2 EDX detector operated at 200 kV. TEM images were obtained using FEI TECNAI T12, operating at 120 kV. The samples for TEM imaging were prepared by depositing the QDs colloidal solution on the carbon‒coated copper TEM grid and evaporating the solvent under inert atmosphere.

## Additional Information

**How to cite this article:** Lin, C.C. *et al*. Luminescent manganese-doped CsPbCl_3_ perovskite quantum dots. *Sci. Rep.* **7**, 45906; doi: 10.1038/srep45906 (2017).

**Publisher's note:** Springer Nature remains neutral with regard to jurisdictional claims in published maps and institutional affiliations.

## Supplementary Material

Supplementary Information

## Figures and Tables

**Table 1:** Lifetimes of QD exciton and Mn^2+^ emission of the CPC:5%Mn‒SiCl_4_ (10 μl) for various reaction times based on a bi‒exponential fit of the luminescence decay curves.

	Lifetime of QDs (ns)			Lifetime of Mn^2+^ (ms)		
Reaction times	τ_1_	τ_2_	τ_avg_	τ_1_	τ_2_	τ_avg_
no SiCl_4_	2.9	9.6	7.5	—	—	—
1 h	3.9	12.1	10.3	0.5	0.2	0.4
1 day	5.1	16.8	15.6	0.2	0.7	0.5
3 days	3.3	9.4	7.5	0.3	1.4	1.3
10 days	2.8	8.1	6.3	0.3	1.4	1.4

## References

[b1] Xing G. . Long‒range balanced electron‒ and hole‒transport lengths in organic‒inorganic CH_3_NH_3_PbI_3_. Science 342, 344‒347 (2013). 24136965 10.1126/science.1243167

[b2] Stranks S. D. . Electron‒hole diffusion lengths exceeding 1 micrometer in an organometal trihalide perovskite absorber. Science 342, 341‒344 (2013). 24136964 10.1126/science.1243982

[b3] Gratzel M. The light and shade of perovskite solar cells. Nat. Mater. 13, 838‒842 (2014). 25141800 10.1038/nmat4065

[b4] Dohner E. R., Jaffe A., Bradshaw L. R. & Karunadasa H. I. Intrinsic white-light emission from layered hybrid perovskites. J. Am. Chem. Soc. 136, 13154‒13157 (2014). 25162937 10.1021/ja507086b

[b5] Li G. . Efficient light‒emitting diodes based on nanocrystalline perovskite in a dielectric polymer matrix. Nano Lett. 15, 2640‒2644 (2015). 25710194 10.1021/acs.nanolett.5b00235

[b6] Sutherland B. R., Hoogland S., Adachi M. M., Wong C. T. O. & Sargent E. H. Conformal organohalide perovskites enable lasing on spherical resonators. ACS Nano 8, 10947‒10952 (2014). 25313937 10.1021/nn504856g

[b7] Zhu H. . Lead halide perovskite nanowire lasers with low lasing thresholds and high quality factors. Nat. Mater. 14, 636‒642 (2015). 25849532 10.1038/nmat4271

[b8] Dou L. . Solution‒processed hybrid perovskite photodetectors with high detectivity. Nat. Commun. 5, 5404 (2014). 25410021 10.1038/ncomms6404

[b9] Maculan G. . CH_3_NH_3_PbCl_3_ single crystals: inverse temperature crystallization and visible‒blind UV‒photodetector. J. Phys. Chem. Lett. 6, 3781‒3786 (2015). 26722870 10.1021/acs.jpclett.5b01666

[b10] Park N. G. Organometal perovskite light absorbers toward a 20% efficiency low‒cost solid‒state mesoscopic solar cell. J. Phys. Chem. Lett. 4, 2423‒2429 (2013).

[b11] Zhou H. . Interface engineering of highly efficient perovskite solar cells. Science 345, 542‒546 (2014). 25082698 10.1126/science.1254050

[b12] Yang Z. . Colloidal quantum dot photovoltaics enhanced by perovskite shelling. Nano Lett. 15, 7539–7543 (2015). 26439147 10.1021/acs.nanolett.5b03271

[b13] Protesescu L. . Nanocrystals of cesium lead halide perovskites (CsPbX_3_, X = Cl, Br, and I): novel optoelectronic materials showing bright emission with wide color gamut. Nano Lett. 15, 3692‒3696 (2015). 25633588 10.1021/nl5048779 PMC4462997

[b14] Nedelcu G. . Fast anion‒exchange in highly luminescent nanocrystals of cesium lead halide perovskites (CsPbX_3_, X = Cl, Br, I). Nano Lett. 15, 5635‒5640 (2015). 26207728 10.1021/acs.nanolett.5b02404 PMC4538456

[b15] Swarnkar A. . Colloidal CsPbBr_3_ perovskite nanocrystals: luminescence beyond traditional quantum dots. Angew. Chem. Int. Ed. 54, 15424–15428 (2015). 10.1002/anie.201508276 26546495

[b16] Nikl M. . Quantum size effect in the excitonic luminescence of CsPbX_3_‒like quantum dots in CsX (X = Cl, Br) single crystal host. J. Lumin. 72‒4, 377‒379 (1997).

[b17] Beaulac R., Ochsenbein S. T. & Gamelin D. R. Colloidal transition‒metal‒doped quantum dots. In nanocrystal quantum dots; Klimov V. I., ed.; CRC Press: New York, 397–453 (2010).

[b18] Beaulac R., Archer P. I., Ochsenbein S. T. & Gamelin D. R. Mn^2+^‒doped CdSe quantum dots: new inorganic materials for spin‒electronics and spin‒photonics. Adv. Funct. Mater. 18, 3873–3891 (2008).

[b19] Bhattacharyya S., Zitoun D. & Gedanken A. Electron paramagnetic resonance spectroscopic investigation of manganese doping in ZnL (L = O, S, Se, Te) nanocrystals. Nanosci. Nanotechnol. Lett. 3, 541–549 (2011).

[b20] Stefan M., Nistor S. V. & Ghica D. ZnS and ZnO semiconductor nanoparticles doped with Mn^2+^ ions. Size effects investigated by EPR spectroscopy. In size effects in nanostructures. Kuncser V., Miu L., eds; Springer Series in Materials Science, Vol. 205, Part 1; Springer: Berlin, Heidelberg, 3–27 (2014).

[b21] Baranov P. G., Orlinskii S. B., De Mello Donega C. & Schmidt J. High‒frequency EPR, ESE, and ENDOR spectroscopy of Co‒ and Mn‒doped ZnO quantum dots. Phys. Status Solidi B 250, 2137–2140 (2013).

[b22] Jana S., Manna G., Srivastava B. B. & Pradhan N. Tuning the emission colors of semiconductor nanocrystals beyond their bandgap tunability: all in the dope. Small 9, 3753–3758 (2013). 23794473 10.1002/smll.201300635

[b23] Erwin S. C. . Doping semiconductor nanocrystals. Nature 436, 91–94 (2005). 16001066 10.1038/nature03832

[b24] Beaulac R., Archer P. I., van Rijssel J., Meijerink A. & Gamelin D. R. Exciton storage by Mn^2+^ in colloidal Mn^2+^‒doped CdSe quantum dots. Nano Lett. 8, 2949–2953 (2008). 18698724 10.1021/nl801847e

[b25] Erickson C. S. . Zero‒reabsorption doped‒nanocrystal luminescent solar concentrators. ACS Nano 8, 3461–3467 (2014). 24621014 10.1021/nn406360w

[b26] Suyver J. F., Wuister S. F., Kelly J. J. & Meijerink A. Synthesis and photoluminescence of nanocrystalline ZnS:Mn^2+^. Nano Lett. 1, 429‒433 (2001).

[b27] Vlaskin Vladimir A., Barrows Charles J., Erickson Christian S. & Gamelin D. R. Nanocrystal diffusion doping. J. Am. Chem. Soc. 135, 14380–14389 (2013). 24028655 10.1021/ja4072207

[b28] Parobek D. . Exciton‒to‒dopant energy transfer in Mn‒doped cesium lead halide perovskite nanocrystals. Nano Lett. 16, 7376–7380 (2016). 27797528 10.1021/acs.nanolett.6b02772

[b29] Liu W. Y. . Mn^2+^‒doped lead halide perovskite nanocrystals with dual‒color emission controlled by halide content. J. Am. Chem. Soc. 138, 14954–14961 (2016). 27756131 10.1021/jacs.6b08085

[b30] Kim Y. . Efficient luminescence from perovskite quantum dot solids. ACS Appl. Mater. Interfaces 7, 25007–25013 (2015). 26529572 10.1021/acsami.5b09084

[b31] Huang S. . Enhancing the stability of CH_3_NH_3_PbBr_3_ quantum dots by embedding in silica spheres derived from tetramethyl orthosilicate in “waterless” toluene. J. Am. Chem. Soc. 138, 5749–5752 (2016). 27100461 10.1021/jacs.5b13101

[b32] Zhang T. . Cellular effect of high doses of silica‒coated quantum dot profiled with high throughput gene expression analysis and high content cellomics measurements. Nano Lett. 6, 800–808 (2006). 16608287 10.1021/nl0603350 PMC2730586

[b33] Jun S., Lee J. & Jang E. Highly luminescent and photostable quantum dot–silica monolith and its application to light‒emitting diodes. ACS Nano 7, 1472–1477 (2013). 23363407 10.1021/nn3052428

[b34] Fardad M. A. Catalysts and the structure of SiO_2_ sol-gel films. J. Mater. Sci. 35, 1835–1841 (2000).

[b35] Ibrahim I. A. M., Zikry A. A. F. & Sharaf M. A. Preparation of spherical silica nanoparticles: Stober silica. J. Am. Sci. 6, 985–989 (2010).

[b36] Ramírez‒Serrano J., Madrigal E., Ramos F. & Caldiño Garcia U. Optical spectroscopy of Mn^2+^ ions in CdCl_2_ single crystals. J. Lumin. 71, 169–175 (1997).

[b37] Marco de Lucas M. C., Rodri´guez F., Gu¨del H. U. & Furer N. Optical properties of the MnCl^4‒^_6_ complex formed in ABCl_3_:Mn^2+^ pseudoperovskite crystals: Influence of the chemical pressure. J. Lumin. 60, 581–584 (1994).

[b38] Zhang W. . Enhanced optoelectronic quality of perovskite thin films with hypophosphorous acid for planar heterojunction solar cells. Nat. Commun. 6, 10030 (2015). 26615763 10.1038/ncomms10030 PMC4674686

[b39] Trizio L. D. & Manna L. Forging colloidal nanostructures via cation exchange reactions. Chem. Rev. 116, 10852–10887 (2016). 26891471 10.1021/acs.chemrev.5b00739 PMC5043423

[b40] Noh J. H., Im S. H., Heo J. H., Mandal T. N. & Seok S. I. Chemical management for colorful, efficient, and stable inorganic-organic hybrid nanostructured solar cells. Nano Lett. 13, 1764–1769 (2013). 23517331 10.1021/nl400349b

[b41] Smith I. C., Hoke E. T., Solis‒Ibarra D., McGehee M. D. & Karunadasa H. I. A layered hybrid perovskite solar‒cell absorber with enhanced moisture stability. Angew. Chem. Int. Ed. 53, 1–5 (2014). 10.1002/anie.201406466 25196933

[b42] Luo B. . Organolead halide perovskite nanocrystals: branched capping ligands control crystal size and stability. Angew. Chem. Int. Ed. 55, 8864–8868 (2016). 10.1002/anie.201602236 27294890

[b43] Midorikawa M., Ishibashi Y. & Takagi Y. Optical and dilatometric studies of KCaCl_3_ and RbCaCl_3_ Crystals. J. Phys. Soc. Japan 46, 1240–1244 (1979).

