# Supplementary material for: Luminescent manganese-doped CsPbCl3 perovskite quantum dots
Source: Sci Rep. 2017 Apr 12;7:45906. doi: 10.1038/srep45906 (PMC5388844; doi:10.1038/srep45906)
Supplement: Supplementary Information [file srep45906-s1.doc]

**Supporting Information**

**Luminescent manganese-doped CsPbCl_3_ perovskite quantum dots**

Chun Che Lin^1^, Kun Yuan Xu^1^, Da Wang^2^ and Andries Meijerink^1^

^1^Condensed Matter and Interfaces, Debye Institute for Nanomaterials Science, Utrecht University, Princetonplein 5, 3584 CC Utrecht, The Netherlands

^2^Soft Condensed Matter, Debye Institute for Nanomaterials Science, Utrecht University, Princetonplein 5, 3584 CC Utrecht, The Netherlands

**Figure S1.** **XRD and PL of bulk CsPbCl_3_:3%Mn^2+^.** (**a**) XRD pattern of the bulk CsPbCl_3_:3%Mn^2+^ (top) compared to the standard powder diffraction pattern (bottom) of cubic phase CsPbCl_3_ (* labels the XRD aluminum stage). (**b**) PL spectrum of the bulk CsPbCl_3_:3%Mn^2+^ under 355 nm excitation (# symbols an instrumental artefact). Inset: optical image of the as**‒**prepared CsPbCl_3_:3%Mn^2+^ powder under UV lamp excitation (λ = 365 nm).

Additional confirmation for the incorporation of Mn^2+^ ions into CPC perovskite QDs is obtained by comparison with the emission of bulk CsPbCl_3_:3%Mn^2+^, shown in Figure S1. The crystal structure of the bulk perovskite synthesized is the cubic phase which is the same as for the QDs (Figure S1a). The Mn^2+^ emission spectrum in the bulk material is similar to that of the QDs, as shown in Figure S1b and Figure 2b (blue line). It indicates that Mn^2+^ ions have been successfully doped into the CPC perovskite QDs.


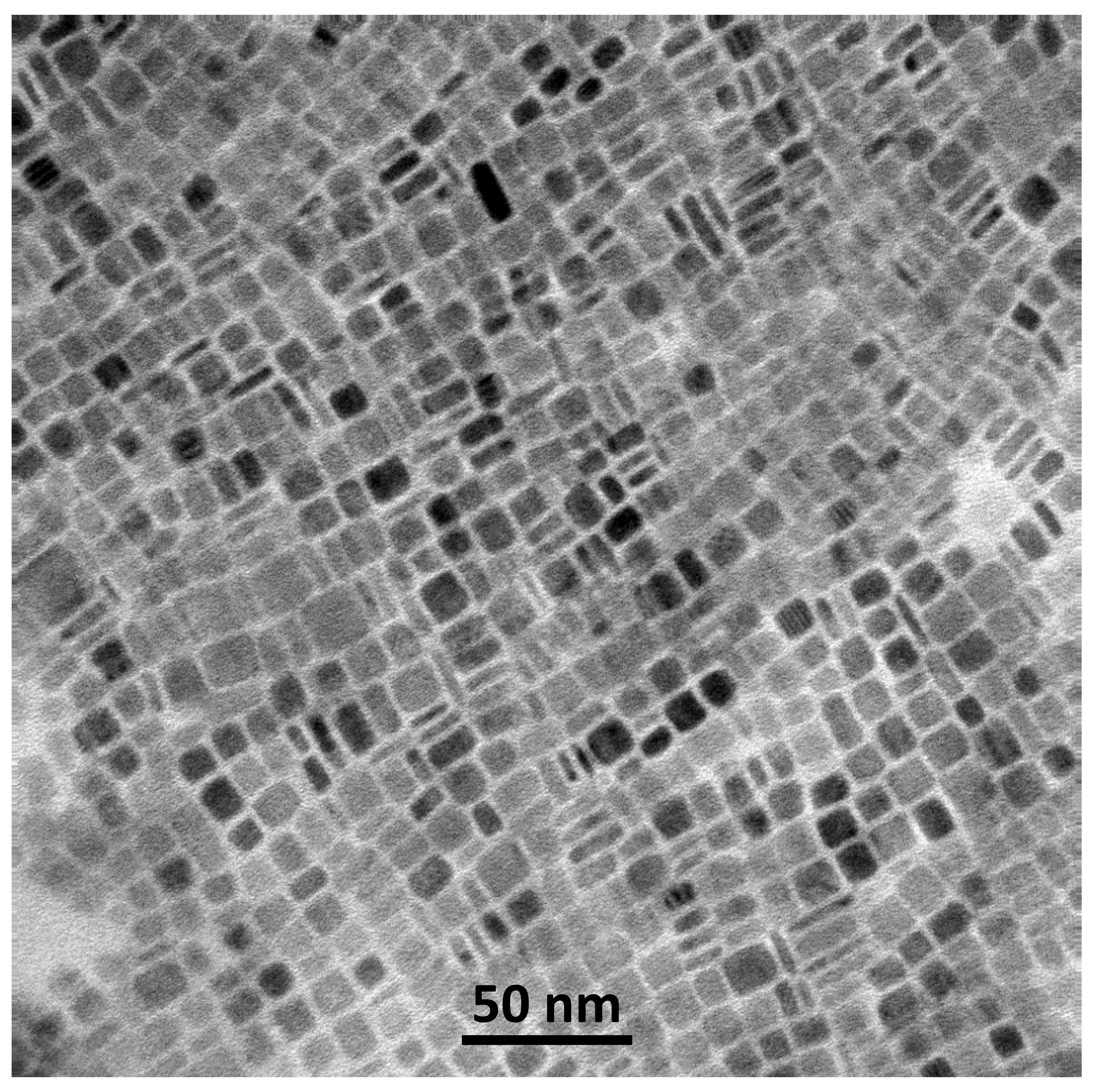


**Figure S2. TEM image of the undoped CsPbCl_3_ QDs.**


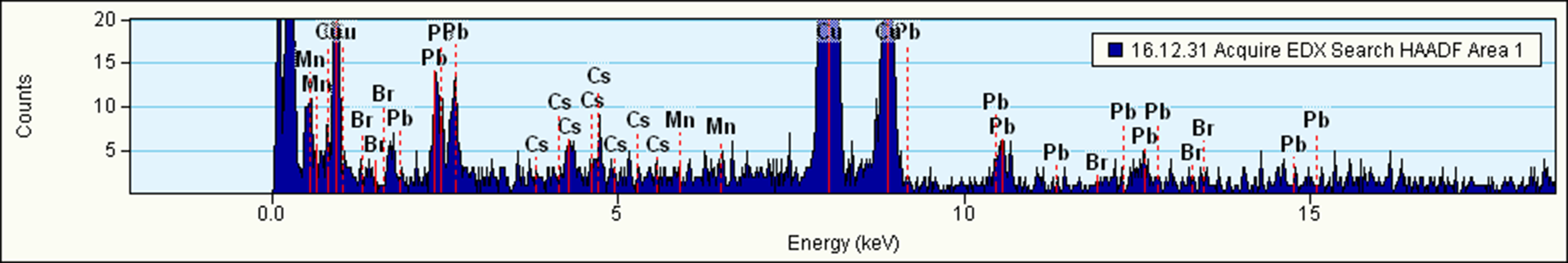


**Figure S3. EDX analysis of the CPC‒Mn QDs.**

**Figure S4. HAADF image (top left) and EDS mapping (in STEM mode) of Cs (red), Pb (green), Cl (blue) and Mn (light blue) in the CPC‒Mn QDs (after centrifuging) for samples made with SiCl_4_ addition followed by stirring 10 days.** In the image on the right hand bottom, the merged elemental mapping reveals that the white dots observed in the HAADF (white circles) are highly enriched in Pb (green).


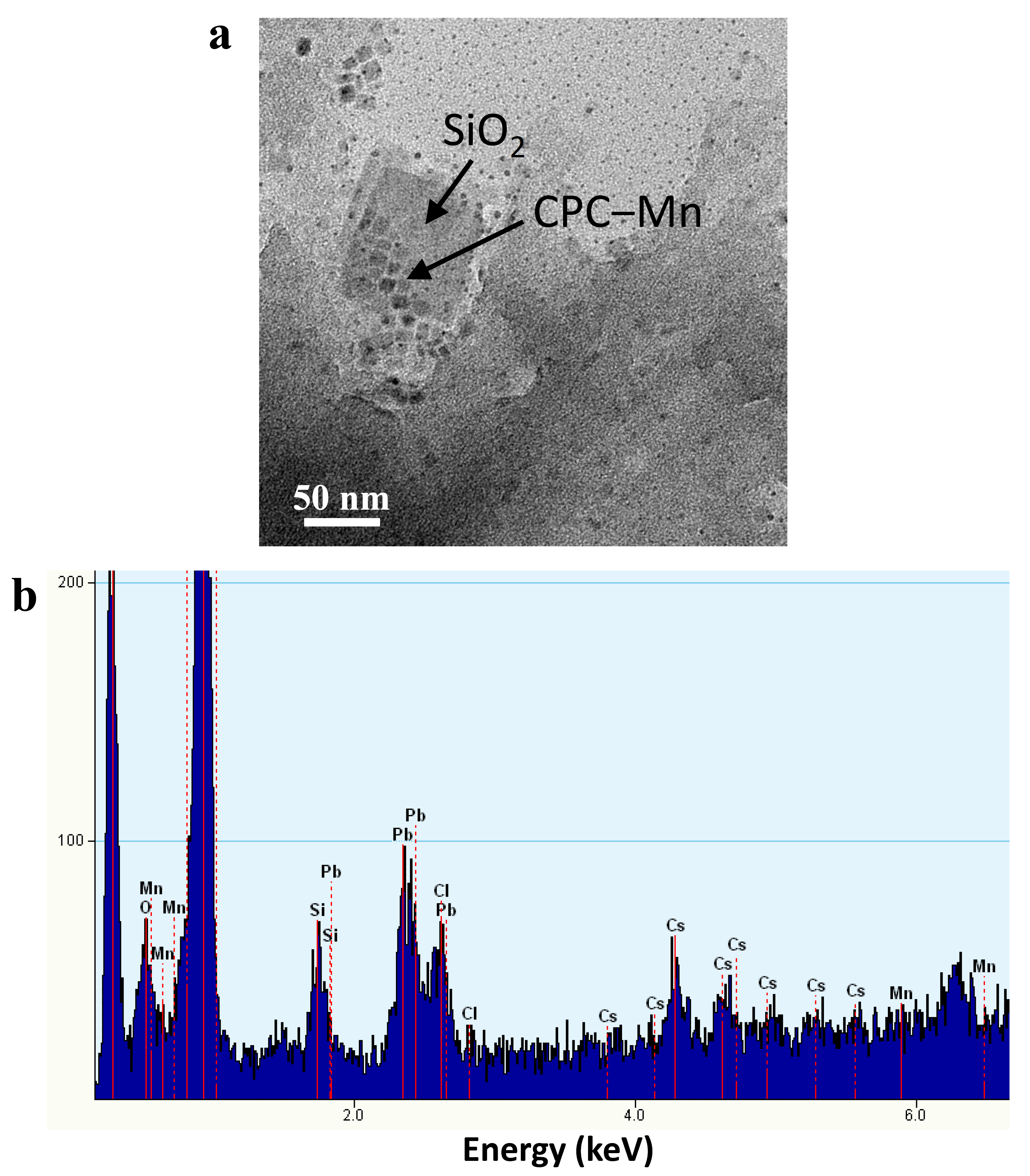


**Figure S5. (a) TEM and (b) EDX analysis of the CPC‒Mn QDs obtained after reaction with SiCl_4_ in vacuum.**


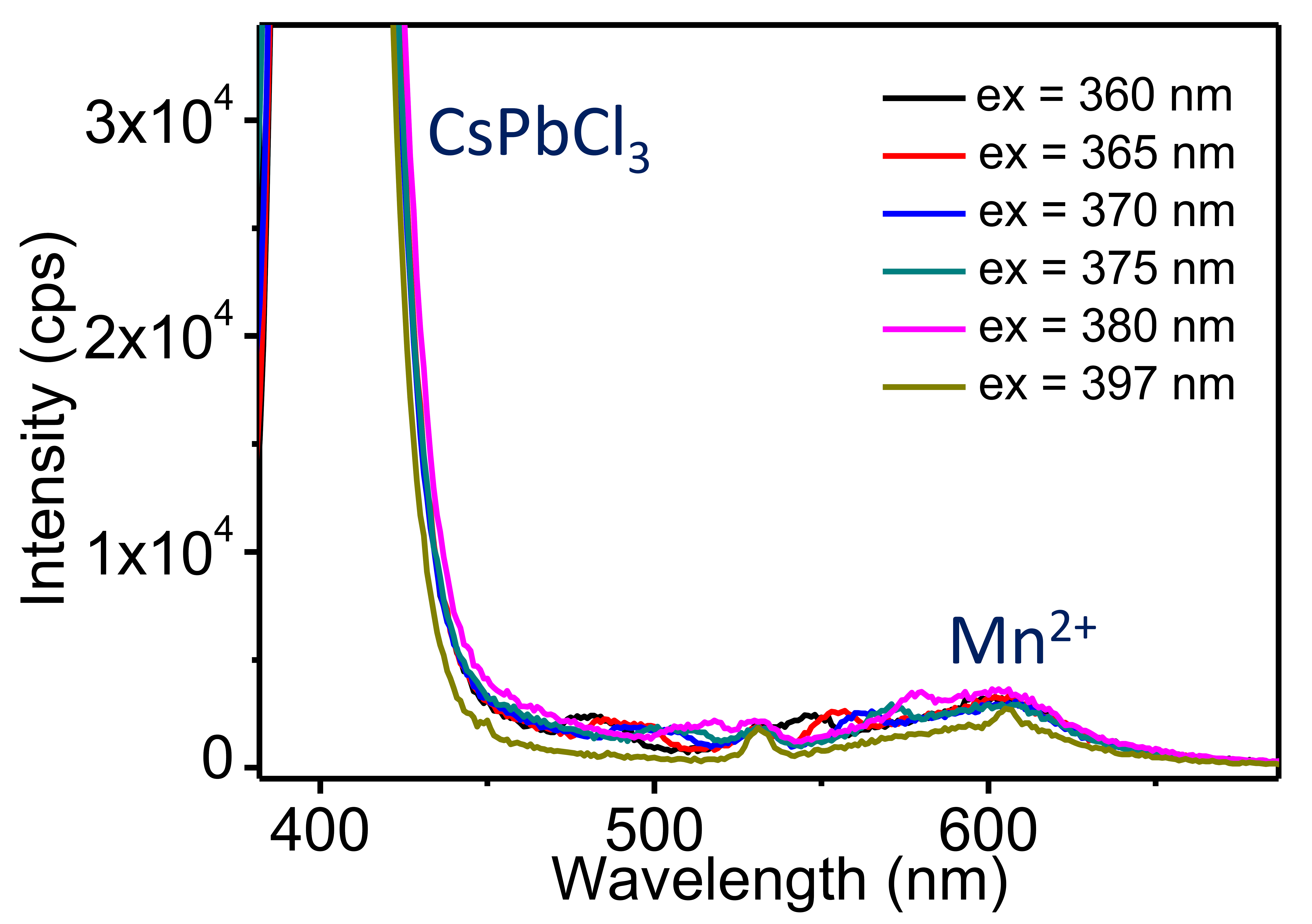


**Figure S6. Enlarged emission spectrum of the CsPbCl_3_:5%Mn^2+^ QDs for various excitation wavelength.** The exciton emission intensity of CPC QDs is around 4 × 10^5^ (cps), whereas the Mn^2+^ emission intensity is lower than 1 × 10^4^ (cps). This result shows that Mn^2+^ precursor is adsorbed in the QD surface layer. Therefore, the energy transfer from the CPC QDs to Mn^2+^ ions is not efficient.

**Figure S7. Emission spectra of the acetone‒washed CsPbCl_3_:5%Mn^2+^ QDs with SiCl_4_ under ambient conditions.**

**Figure S8. Emission spectrum of the CsPbCl_3_:5%Mn^2+^ QDs upon addition of different amounts (0 to 20 μl) of SiCl_4_ under ambient conditions.** (**a**) The inset shows a photograph of undoped CsPbCl_3_ (1), CsPbCl_3_:5%Mn^2+^ (2), CsPbCl_3_:5%Mn^2+^ with SiCl_4_ (3) and decomposed CsPbCl_3_ after addition of 20 μl of SiCl_4_ (4) colloidal solutions in toluene under UV lamp excitation (λ = 365 nm). (**b**) Integrated intensity of QD exciton and Mn^2+^ d‒d emission after addition of different amounts of SiCl_4_.


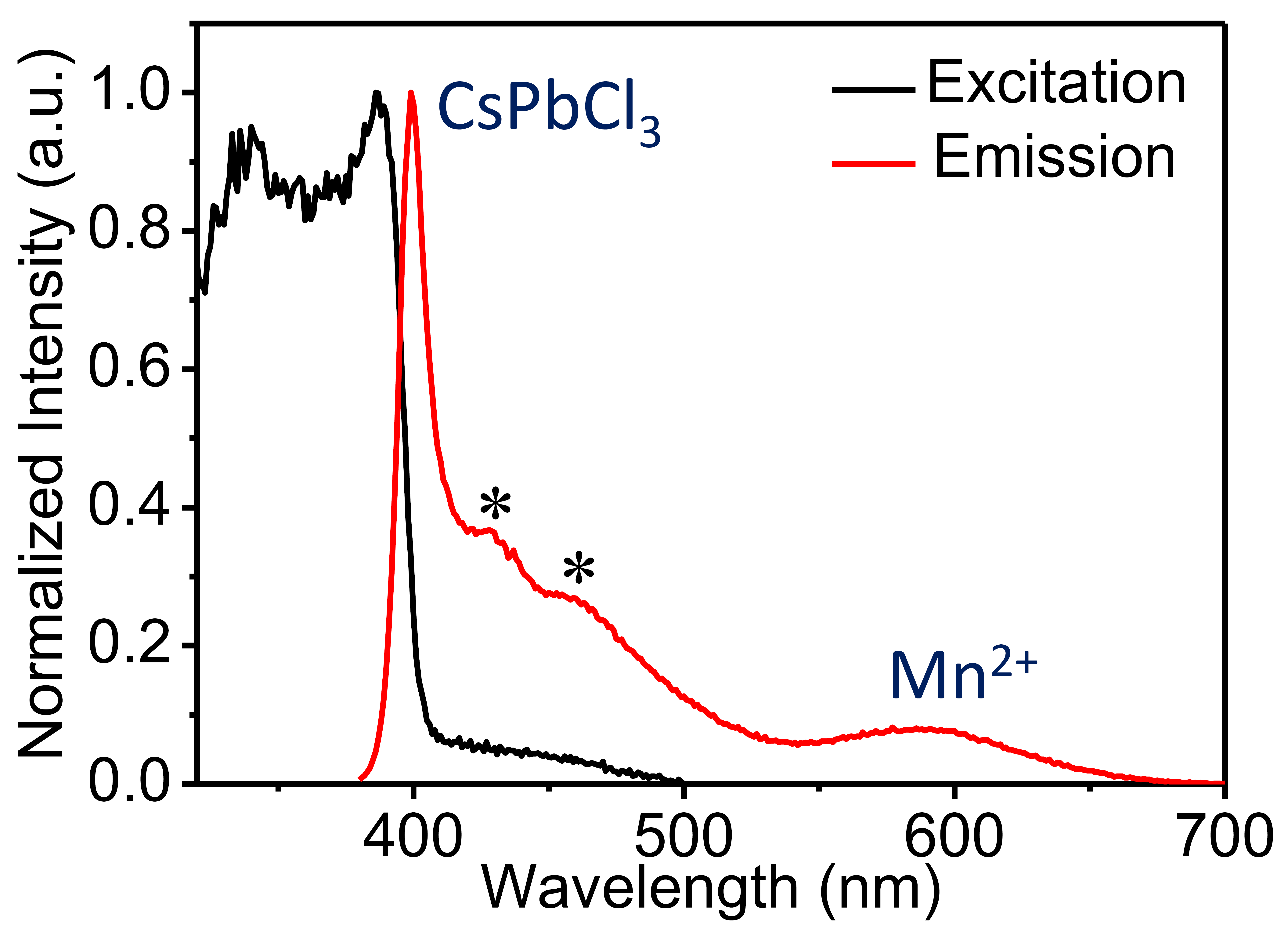


**Figure S9. Excitation and emission spectra of the CsPbCl_3_:5%Mn^2+^ QDs after SiCl_4_ addition in vacuum stirred for one month.** In addition to QDs and Mn^2+^ emission (red line), the symbol (*) marks emission in the blue spectral region that is assigned decomposed ligands. The excitation spectrum (black line) of the Mn^2+^ emission was measured at 600 nm.
